# Addition of defatted green‐lipped mussel powder and mixed spices to wheat–purple rice flour biscuits: Physicochemical, in vitro digestibility and sensory evaluation

**DOI:** 10.1002/fsn3.675

**Published:** 2018-08-17

**Authors:** Warinporn Klunklin, Geoffrey Savage

**Affiliations:** ^1^ Faculty of Agriculture and Life Sciences Lincoln University Lincoln, Christchurch New Zealand

**Keywords:** antioxidants, biscuit, green‐lipped mussel powder, in vitro digestion, purple rice flour, sensory evaluation

## Abstract

Biscuits were formulated using a 50/50 wheat and purple rice flour mix containing mixed spices, galangal, and defatted green‐lipped mussel powder (*Perna canaliculus*) added in the range of 5–15% of the total biscuit weight. The fortified biscuits had higher protein (26.36%), fiber (52.90%), and ash (6.00%) contents and a lower total fat (5.64%) content compared to the control biscuits. The in vitro starch digestibility and predicted glycemic index (pGI) decreased in the fortified biscuits by 18.95% and 6.18%, respectively, while the in vitro protein digestibility increased by 3.73%, corresponding to the increased levels of defatted mussel powder present. The spread ratio and hardness of the fortified biscuits also increased significantly. The color values of the fortified biscuits after the incorporation of different levels of defatted mussel powder showed significant changes, with a darkening of the biscuit surface and a lowered browning index compared to the control biscuits. Results of the sensory quality evaluation showed that incorporation of defatted mussel powder into the biscuit mix of up to 15% showed no significant differences in liking scores in terms of color, overall appearance; whereas, the flavor and overall acceptability scores were significantly lower than the control biscuits. Overall, defatted mussel powder can be successfully incorporated into biscuit mixes to enrich the protein, fiber, and antioxidant contents of the biscuits.

## 1 INTRODUCTION

Many agricultural crops, such as flaxseeds and garden cress seeds, have been used as natural sources of bioactive compounds that meet the demands of health conscious consumers (Kaur, Singh, & Kaur, 2017; Umesha, Manohar, Indiramma, Akshitha, & Naidu, 2015). *Oryza sativa* L. Var. Sanpatong, purple rice, widely grown in Northern Thailand, is becoming a popular crop because of its improved taste, lower price, and high levels of antioxidants (i.e., anthocyanins and phenolic acids) compared to wheat flour (Jang & Xu, 2009). Anthocyanins (cyanidin‐3‐o‐glucoside and peonidin‐3‐o‐glucoside) are present in the aleurone layers and pericarp of the rice, while phenolic acids are in the outer layers of the rice grain and these have potential antioxidant activities (Jang & Xu, 2009). In addition, purple rice also contains very high dietary fiber levels (Das, Goud, & Das, 2017; Jang & Xu, 2009; Yawadio, Tanimori, & Morita, 2007). Earlier studies by Klunklin and Savage (2018) have reported that purple rice flour is a good ingredient for making biscuits and also contains high levels of antioxidants.

Improvement in the protein contents of biscuits has been undertaken previously by the incorporation of whey protein, rice flour protein and soy protein (Mancebo, Rodriguez, & Gomez, 2016; Šaponjac et al., 2016; Wani, Gull, Allaie, & Safapuri, 2015). Green‐lipped mussel (*Perna canaliculus*) powder is widely known as a dietary supplement and contains bioactive components that are rich in polyunsaturated fatty acids. However, there is a limit to the use of mussel powder in food products due to problems with lipid oxidation resulting in the development of undesirable odors and taste (Umesha et al., 2015). Defatted mussel powder has had the oil fraction removed to produce mussel products for the human supplement market, but still contains some residual fatty acids (Vijaykrishnaraj, Kumar, & Prabhasankar, 2015; Vijaykrishnaraj, Roopa, & Prabhasankar, 2016). The defatted mussel powder can be used to enrich the protein in food products (Vijaykrishnaraj et al., 2016). A few studies have evaluated the addition of different levels of green‐lipped mussel powder in pasta and bread (Vijaykrishnaraj et al., 2015, 2016). Addition of green‐lipped mussel is the cause of high off‐flavors in the final product due to lipid oxidation (Vijaykrishnaraj et al., 2015).

Natural antioxidants are important alternative ingredients that can be used to protect the oil from oxidation during thermal processing (Zhang et al., 2013); they are also able to protect the free radicals present (Pasqualone et al., 2015). Natural antioxidant compounds can be found in fruits, vegetables and spices, and also in various types of pigmented cereals, such as red, purple, and black rice or wheat grains (Pasqualone et al., 2015; Yawadio et al., 2007). Since ancient times, spices have been used as basic ingredients in Southeast Asian cuisine, and throughout the world, to develop the flavor of foods (Su et al., 2007). Many studies have researched the oxidative stability and antioxidant contents of culinary spices in food products due to their growing use as natural dietary sources (Juntachote, Berghofer, Siebenhandl, & Bauer, 2007; Misan et al., 2011; Ramanathan & Das, 1993; Ruangchakpet & Sajjaanantakul, 2007; Su et al., 2007; Umesha et al., 2015). These studies reported that culinary spices contain antioxidant compounds with effective antioxidant activities. Nutmeg and cinnamon are widely used in food products as flavoring ingredients with the benefits of reducing lipid oxidation and assisting human health by controlling diabetes, as well as having antimicrobial and anti‐inflammatory effects (Su et al., 2007).

In the baking industry, the application of functional foods is an important developing market. Much bakery research has attempted to make healthy products by incorporating new ingredients into biscuit mixes in order to increase the nutritional qualities of the final product (Giarnetti, Paradiso, Caponio, Summo, & Pasqualone, 2015; Misan et al., 2011; Park, Choi, & Kim, 2015; Sozer, Cicerelli, Heiniö, & Poutanen, 2014; Umesha et al., 2015). Biscuits are largely consumed around the world due to their affordable price, good taste, and long shelf life (Park et al., 2015). Biscuits generally contain high carbohydrate, sugar and fat contents. From a previous study, it was shown that purple rice flour could be substituted for some of the refined wheat flour to make bakery products, such as biscuits. (Klunklin & Savage, 2018). These biscuits had low starch digestibility, low pGI, and high protein digestibility, with only small changes in the physical characteristics of the biscuits. A 50% purple rice flour substitution was accepted by most of the panelists who tasted the experimental biscuits (Klunklin & Savage, 2018). In a previous study (Klunklin & Savage, 2018), the mussel biscuits were fortified by adding ginger and galangal to cover up the slight fishy smell and increase the antioxidant content at the same time. For this study, biscuits were supplemented using wheat–purple rice blended flour containing mixed spices, such as cinnamon, nutmeg, and galangal in order to increase the liking scores of the fortified biscuits tested by untrained consumers. The biscuits were then enriched with different levels of defatted mussel powder that improved the physicochemical, antioxidant properties and sensory evaluation of the fortified biscuits.

The research objective was to evaluate the physicochemical characteristics, antioxidant properties and consumer preferences of fortified biscuits enriched with different levels of defatted green‐lipped mussel powder, ranging from 5% to 15% of the total biscuit mix with the added mixed spices.

## 2 MATERIALS AND METHODS

### 2.1 Raw materials

Biscuits were prepared using refined wheat flour (Pams Products Ltd., Auckland, New Zealand), whole purple rice grains (*Oryza sativa* L. Var. Sanpatong) (Big T. supermarket, Riccarton, Christchurch, New Zealand) imported in 5‐kg bags, baking powder (Edmonds Limited, Goodman Fielder Ltd., Auckland, New Zealand), xanthan gum (Lotus foods Pty Ltd., Cheltenham, Victoria, Australia), butter (Dairyworks Ltd., Hornby, Christchurch, New Zealand), castor sugar (Pams Products Ltd.), salt (Pams Products Ltd., Auckland, New Zealand), whole egg (Pams Products Ltd.), vanilla extract (Pams Products Ltd.), mixed spices nutmeg and cinnamon in a 2:1:1 ratio (Cerebos Gregg's Limited, Christchurch, New Zealand), galangal powder (Sunson gifts and the Asian food market, Riccarton), and defatted green‐lipped mussel powder (*Perna canaliculus*) provided by Aroma New Zealand Limited, Christchurch, New Zealand. All ingredients were food grade and all chemicals were analytical grade. The experimental biscuits were prepared by the addition of mussel powders at levels of 5, 10, and 15% (w/w) of the total flour weight.

### 2.2 Biscuit preparation

The experimental biscuits were prepared using a 50/50 mix of refined wheat flour and whole purple rice flour. The purple rice grains were milled using a Whisper Mill (Grote Molen Inc., USA). All ingredients are shown in Table 1. The biscuit dough was rolled to a thickness of 4 mm using an adjustable rolling pin (Joseph Joseph 20085; Joseph Joseph Ltd., London, UK). The dough was cut to round shapes using a 5‐cm‐diameter biscuit cutter and then baked at 170°C for 9 min in the oven (Bakbar Turbofan 32 Max; Ali group company, Milan, Italy). The biscuits were cooled at room temperature for 15 min before they were packed in sealed aluminum bags.

**Table 1:** Formulation of different biscuits supplemented with defatted mussel powder

Ingredients	Experimental biscuits (g)	
	Control	Fortified biscuit
Refined wheat flour	50	50
Purple rice flour	50	50
Defatted mussel powder	–	Varied from 5%–15%
Butter	75	75
Sugar	62.5	62.5
Egg	112.5	112.5
Salt	0.75	0.75
Mixed spices	–	2
Cinnamon	–	1
Nutmeg	–	1
Galangal	–	4
Sodium bicarbonate	4.5	4.5
Xanthan gum	1.25	1.25

### 2.3 Proximate analysis

All biscuit samples were analyzed in triplicate. The moisture content was determined using the oven drying method at 105°C for 18 hr (AOAC, 2002). The total nitrogen was evaluated using the Dumas method by rapid Max N Exceed^®^ (Elementar, Hanau, Germany) and a factor of 6.25 was used to estimate the protein contents. The fat and ash contents of the biscuits were determined using standard methods 920.176 and 900.029 respectively (Association of Official Agricultural Chemistry, 2002). Total carbohydrate was calculated by difference (AOAC, 2000) and total dietary fiber (TDF) was measured using a total dietary fiber assay kit (Sigma‐Aldrich, MO, USA). The total starch content was determined using a Megazyme starch assay kit (Megazyme International Ireland Ltd., Wicklow, Ireland), using approved method 76–13 (Reed, Ai, Leutcher, & Jane, 2013).

### 2.4 In vitro digestibility

#### 2.4.1 Protein digestibility

The in vitro protein digestibility was determined according to the method of Akeson and Stahmann (1964). The enzymes used were pepsin from porcine gastric mucosa (Sigma Aldrich, USA, 66 units/mg protein) followed by pancreatin from porcine pancreas (AppliChem Chemica Synthesis, Germany, 30.315 units/mg protein). All simulated solutions from the digestibility were centrifuged at 1600 *g* for 10 min. The supernatants were measured using an Elementar Vario TOC cube instrument fitted with a chemiluminescence detector to determine total bound nitrogen (TNb) to determine the digested protein. The analysis was carried out in triplicate.

#### 2.4.2 Starch digestibility

The available starch contents were assessed following the multienzymatic protocol of Monro, Mishra, and Venn (2010) using porcine pepsin (Sigma Aldrich), porcine pancreatic alpha‐amylase (No. 7545, Sigma–Aldrich, St. Louis, MO), and amyloglucosidase (No. 9913, Sigma–Aldrich). The enzyme solutions were made fresh before each analysis. The digested solutions were centrifuged at 180 *g* for 5 min. The supernatants were read at 530 nm (V‐1200 spectrophotometer, Global Science, Auckland, New Zealand).

### 2.5 Predicted glycemic index (pGI)

The hydrolysis index (HI) was calculated from the area under the curve (AUC) of each biscuit using the in vitro starch digestibility results divided by the AUC of a standard (50 g white bread). The predicted glycemic index (pGI) was calculated according to the method of Goñi, García, and Saura‐Calixto (1997). Predicted glycemic index = 39.71 + 0.549 × HI.

### 2.6 Physical characteristics

All physical analyses of biscuits were measured 15 min after baking. A texture analyser (TA‐XT2i, Stable Micro System, Godalming, UK) equipped with a small 3‐point bending test rig with a sharp‐blade cutting probe and with a 30‐kg load cell (Park et al., 2015). Hardness measurements, expressed as the first peak force of each penetration, were carried out. Six biscuits were weighed and their width (W), thickness (T), and biscuit spread ratio (W/T) were measured using AACC method 10‐50.05 (AACC, 1967; AACC, 2000) .

### 2.7 Color determination

Surface CIE color values (L*a*b*) were determined using a Minolta Chroma Meter CR‐410 (Konica Minolta, Chiyoda, Tokyo, Japan) and the results were presented in the CIE L* a* b* system in which L* represented lightness, a* represented redness, while b* represented yellowness (Sozer et al., 2014). The browning indexes of the biscuits were calculated as reported by Ruangchakpet and Sajjaanantakul (2007). All analyses were performed in triplicate.

### 2.8 Antioxidant compounds and antioxidant activity determinations

Biscuits were extracted following the protocol of Jang and Xu (2009) to obtain both lipophilic and hydrophilic antioxidants. The extracted solutions were kept at −20°C until further analysis. The total phenolic contents in the extracted solutions were measured according to the method of Kaneda, Kubo, and Sakurai (2006). The total flavonoid content was determined according to the procedure used by Zhishen, Mengcheng, and Jianming (1999). The anthocyanin content was determined according to Hosseinian, Li, and Beta (2008).

The DPPH scavenging capacity was undertaken according to the method of Mahakunakorn, Tohda, Murakami, Matsumoto, and Watanabe (2004). The ABTS assay was evaluated using the method of Re et al. (1999). All analyses were determined in triplicate.

### 2.9 Sensory evaluation

One hundred and seven panelists, aged between 19 and 60 years, evaluated the biscuits in terms of overall appearance, touch/oiliness, crunchiness, flavor, and overall acceptability. The panelists were untrained students and staff members from Lincoln University. The sensory evaluation was approved by the Lincoln University Human Ethics Committee No. 2017‐24. All panelists signed the consent form prior to being served the samples. A 9‐point hedonic scale (1 =  dislike extremely, 9 =  like extremely) was used to assess the liking and disliking scores of the panelists (Aminah & Tan, 2001). Before consuming the biscuits, panelists were asked to evaluate the overall appearance and touch/oiliness using a 9‐point hedonic scale ranging from “like extremely” to “dislike extremely”. After scoring the overall appearance, the panelists were then allowed to evaluate the crunchiness, flavor, and overall acceptability of the biscuits. The evaluation of samples was completely randomized and performed 1 day after baking with. The sensory analysis was carried out in a standardized sensory analysis room.

### 2.10 Statistical analysis

The experimental data were statistically analyzed by one‐way ANOVA using a complete randomized design for all physicochemical experiments, and a randomized complete block design for sensory analysis followed by Duncan's multiple range test using SPSS Statistics (v. 22.0, SPSS Inc., Chicago, IL, USA) at *p < *.05 to determine the significant differences. The least significant differences (LSD) were analyzed with Minitab (v.17, Minitab Inc., State College, PA, USA).

## 3 RESULTS AND DISCUSSION

### 3.1 Nutritional compositions

The chemical compositions of the biscuits prepared using 5%, 10%, and 15% of defatted green‐lipped mussel powder are shown in Table 2. The moisture content in the biscuits decreased significantly (*p < *.05) with increased mussel powder incorporation, from 5% to 15%. In general, biscuits were considered to be very low moisture content products. The moisture content of the biscuits normally ranged from 1% to 5%, which prevents microbiological spoilage and extends the shelf life of products (Chung, Cho, & Lim, 2014). Chung et al. (2014) reported that the fortification of biscuits with increasing fiber and protein contents affected the moisture content of the biscuits. Therefore, the biscuits prepared with the addition of defatted mussel powder had a low water retention capacity in the biscuit matrix compared to the control biscuits. This might have contributed to a change in moisture content during baking. Increasing the mussel powder content in the biscuit mix (Table 2) had the greatest effect on the crude protein content. All biscuits supplemented with defatted mussel powder were found to be nutritious snacks. The consumption of 100 g of defatted mussel biscuits would provide more than half of the recommended daily protein requirements for children and young people aged between 5 and 19 years (25–30 g/day) according to the FAO/WHO (1973). This suggests that these biscuits enriched with defatted mussel powder would be useful as protein‐supplemented snacks in developing countries and could be used as a supplement for people who are unable to consume meat protein.

**Table 2:** Basic characteristics of the fortified biscuits prepared from wheat–purple rice flour blends mixed with mussel powder and mixed spices

Parameters (% DM basis)	% defatted green‐lipped mussel powder				LSD (5%)
	0 (Control)	5	10	15	
Moisture content (%)	5.06 ± 0.03^a^	4.99 ± 0.05^b^	4.73 ± 0.01^c^	4.69 ± 0.03^d^	0.05
Crude protein (%)	7.31 ± 0.13^d^	8.50 ± 0.04^c^	9.33 ± 0.17^b^	9.88 ± 0.07^a^	0.14
Protein digestibility (%)	82.59 ± 1.33^NS^	85.17 ± 0.06	85.73 ± 0.85	86.12 ± 0.70	3.54
Total dietary fiber (%)	15.76 ± 0.11^d^	20.61 ± 0.30^c^	23.76 ± 0.09^b^	27.45 ± 0.07^a^	0.28
Ash (%)	2.33 ± 0.03^c^	2.27 ± 0.03^bc^	2.47 ± 0.03^b^	2.67 ± 0.07^a^	0.07
Total fat (%)	25.30 ± 0.50^a^	23.37 ± 0.10^b^	24.05 ± 0.27^ab^	24.43 ± 0.20^ab^	0.50
Total starch (%)	33.70 ± 1.79^NS^	36.91 ± 0.37	35.42 ± 1.01	35.05 ± 1.43	3.26
AUC of digested starch (mg min/dl)	462.01 ± 0.43^a^	422.17 ± 0.26^b^	391.67 ± 0.28^c^	351.35 ± 0.75^d^	1.26
pGI	62.56 ± 0.02^a^	60.59 ± 0.01^b^	59.08 ± 0.01^c^	57.09 ± 0.04^d^	0.07

AUC, Area under the curve; LSD, least significant difference; NS, not significant.

Values represent mean ± SE. In each row, sample means not having the same letter are significantly different (Duncan's multiple range test, *p *<* *.05).

The addition of 15% mussel powder in the biscuits resulted in a significant rise in total fiber content. From a nutritional point of view, to claim that a food is a “source of dietary fiber,” it needs to contain at least 3 g/100 g, whereas “high in dietary fiber” foods should contain at least 6 g/100 g according to regulation (EC) 1924/2006 (Official Journal of the European Union, 2006). Therefore, all biscuits in this study can be considered to be high dietary fiber food products. The biscuits enriched with defatted mussel powder contained less fat compared to the control biscuits. A similar trend was reported by Arshad, Anjum, and Zahoor (2007), that the addition of the defatted wheat germ decreased the fat content when the total dietary fiber content of the biscuits was increased. Fat content is one of the basic components of biscuits which has positive effects on texture characteristics such as mouth feel. Similar fat contents (Table 2) have also been observed by Baltsavias, Jurgens, and van Vliet (1999) who reported that fat can vary from 20% to 60% based on percentage by weight of flour used to make the biscuits. However, butter contains high content of saturated fatty acids (Baltsavias et al., 1999; Giarnetti et al., 2015). A further study would investigate the possibility of reducing the total fat content and also the possibility of using a fat with a healthier fatty acid profile (Giarnetti et al., 2015). There were no significant differences (*p > *.05) in total starch contents of all the biscuits.

The overall reduction in starch digestibility was of great interest as starch is a major ingredient in these biscuits. The area under the starch hydrolysis curve (AUC) was evaluated, after the digestion of each biscuit, to determine the glycemic response of the selected foods compared to a reference sample of white bread (An, Bae, Han, Lee, & Lee, 2016). The AUC of the biscuits enriched with defatted mussel powder was significantly lower (*p < *.05), by 26.4%, than the control biscuits. This is the first study of the starch digestibility of defatted mussel powder incorporated within a food product. Lower starch digestibility following the additions of defatted mussel powder resulted from protein–starch interactions, which formed a matrix surrounding the starch granules, which then acted as a physical barrier to limit starch availability (Singh, Dartois, & Kaur, 2010). This result showed that defatted mussel powder can be considered as an important ingredient that was able to reduce a starch digestibility when incorporated into food products.

The pGI was calculated according to Goñi et al. (1997) using the starch hydrolysis index (HI) of each biscuit containing added defatted mussel powder. As expected, including defatted mussel powder as an enriched ingredient caused a reduction in the pGI of the biscuits (Table 2). Brand‐Miller (1994) showed that a diet containing low glycemic index foods reduced insulin resistance and adjusted the blood glucose levels in both Type 1 and Type 2 diabetic patients. These fortified biscuits have a low glycemic index (GI < 60), and would be a good snack food for diabetics. The control biscuits can be regarded as an intermediate GI (60–85) snack.

### 3.2 Biscuit quality

The addition of purple rice flour and defatted mussel powder to a basic biscuit mix influenced the physical characteristics (Table 3). The hardness of the biscuits significantly increased (*p *<* *.05), from 5% to 15%, with the increased amounts of defatted mussel powder, compared to the control biscuits. This could be attributed to the lower levels of wheat gluten available to bind with water as the flour content was decreased by adding more defatted mussel powder into the biscuit mix. Moreover, the moisture content of the biscuits inversely affected the hardness of the fortified biscuits (Mancebo et al., 2016). All these factors gave the biscuits a higher hardness and lower spread ratio. However, the hardness of these experimental biscuits was in the same range as biscuits containing rice flour (Chung et al., 2014).

**Table 3:** Physical, textural, and L*a*b* color characteristics of the fortified biscuits prepared from wheat–purple rice flour blends mixed with mussel powder and mixed spices

Physical parameters	% defatted green‐lipped mussel powder				LSD (5%)
	0 (Control)	5	10	15	
Hardness (N)	19.05 ± 0.07^d^	19.43 ± 0.02^c^	19.95 ± 0.11^b^	20.49 ± 0.06^a^	0.17
Width (W, mm)	62.67 ± 0.21^a^	61.17 ± 0.48^b^	61.00 ± 0.37^b^	60.67 ± 0.33^b^	0.75
Thickness (T, mm)	6.50 ± 0.22^c^	7.17 ± 0.75^bc^	7.50 ± 0.22^b^	8.33 ± 0.82^a^	0.68
Spread ratio (W/T)	9.70 ± 0.33^a^	8.60 ± 0.40^b^	8.20 ± 0.30^bc^	7.35 ± 0.32^c^	0.81
Color values					
L*	78.46 ± 0.13^a^	77.31 ± 0.08^bc^	77.62 ± 0.07^b^	77.06 ± 0.20^c^	0.27
a*	−3.22 ± 0.08^a^	−4.49 ± 0.11^b^	−4.93 ± 0.05^c^	−5.07 ± 0.05^c^	0.15
b*	25.40 ± 0.08^a^	24.04 ± 0.11^c^	24.34 ± 0.12^b^	23.87 ± 0.09^c^	0.21
Browning index	34.82 ± 0.23^a^	31.67 ± 0.21^b^	31.60 ± 0.24^b^	30.87 ± 0.20^c^	0.47

LSD, least significant difference.

Values represent mean ± SE. For each row, sample means not having the same letter are significantly different (Duncan's multiple range test, *p *<* *.05).

The spread ratio is an important measurement of biscuit quality, higher values are considered to be more desirable. The spread ratio of the fortified biscuits showed a significant decrease (*p *<* *.05) with the increased defatted mussel powder addition levels compared to the control biscuits (Table 3). This was the first time that the addition of defatted mussel powder with mixed spices has been investigated in a biscuit mix. Moreover, Vijaykrishnaraj et al. (2016) found that adding green‐lipped mussel hydrolysate to gluten‐free bread affected the physical characteristics, such as the volume and crumb formation. In this study, the width decreased, and the thickness increased after the incorporation of green‐lipped mussel powder into the biscuit mix. The increased level of fiber and protein content from the defatted mussel powder (Table 2) absorbed more water. The water in the biscuit formulation was limited, as a result the biscuits remained harder so, consequently, they had a smaller spread ratio (Chung et al., 2014). Lower dough expansion during baking was also observed by Park et al. (2015) and Pasqualone et al. (2015) in biscuits formulated from alternative flours that contained increased protein content.

Consumers considered buying food products based on their color, and this was an important factor in the acceptance of a new food. The fortified biscuits showed a significant reduction in all L*a*b* color parameters (Table 3). The control biscuits were relatively dark, as they were made from 50% purple rice flour. Color darkening of biscuits during cooking normally occurred from sugar caramelization and/or the Maillard reactions between sugars and amino acids in the mixture (Chung et al., 2014). The addition of protein can also reduce the L* values of the biscuits. Other authors found similar results when they incorporated additional protein into biscuits (Park et al., 2015; Sozer et al., 2014). As the incorporation of protein decreased the a* and b* values of the biscuits, the additional green color of the biscuits resulted from the addition of defatted mussel powder. There were increased negative a* values (greenness) in the fortified biscuits, whereas the b* values of the biscuits decreased, the color moved from yellow to a blue hue due to the presence of the defatted mussel powder (higher in the control biscuits than in the fortified biscuits). Similar results were also reported by Vijaykrishnaraj et al. (2015, 2016) who observed that the addition of green‐lipped mussel powder altered the color values of food products.

The browning index is commonly used to determine the end of the baking process as it was the last step of both the Maillard reaction and caramelization (Torbica, Hadnađev, & Hadnađev, 2012). In this study, the rate of Maillard reaction reduced with the increased incorporation of the defatted mussel powder in biscuit mixes due to reductions in the browning index. The rate at which the sugars present dissolved, depended on the amount of water present in the biscuit dough (Torbica et al., 2012); hence, the water content of the fortified biscuits was lower than in the control biscuits (Table 2). Vijaykrishnaraj et al. (2016) also confirmed that the small molecules produced by the thermal degradation of starch and proteins in the mussel powder had not induced pronounced Maillard reactions during the baking process.

### 3.3 Antioxidant compounds and antioxidant activities

The total phenolic contents of the biscuits significantly increased (*p *<* *.05) as the percentage of mussel powder increased from 5% to 15% in the biscuit mix (Table 4). Many researchers have studied wheat biscuits enriched with polyphenols from different ingredients, such as ginger, gooseberry, flaxseed flour, and purple wheat extract (Abdel‐Samie, Wan, Huang, Chung, & Xu, 2010; Kaur et al., 2017; Pasqualone et al., 2015; Ruangchakpet & Sajjaanantakul, 2007). The contents of phenolic compounds in baked cereal products were normally affected by the moisture content on baking, while the total phenolic compounds of purple rice ranged between 492 and 2013 μg of gallic acid/g DW using the same extraction process as in the study by Jang and Xu (2009). The main anthocyanins of defatted mussel powder biscuits were analyzed in this study. Cyanidin‐3‐o‐glucoside (C3G) significantly increased (*p *<* *.05) with the increased proportion of defatted mussel powder in the biscuit mix. Anthocyanins have been found in high amounts in purple rice (Jang & Xu, 2009) but there has been no research on the anthocyanin content of defatted mussel powder. In addition to the total flavonoids, the phenolic acids had a high positive correlation with the content of total phenolic compounds. Thus, the flavonoid levels were 89.29% higher in the 15% defatted mussel powder biscuits compared to the control biscuits. The antioxidant compounds present depended on the breakdown of phenolics by heat in the food matrix, which was affected by the extraction rates of the phenolics (Jang & Xu, 2009). There have not been any reports about the antioxidant content of defatted green‐lipped mussel powder. The protein isolated from blue mussel (*Mytilus edulis*) and green‐lipped mussel powder (*Perna canaliculus*) also showed significant radical scavenging activity and acted as inhibitors of auto‐oxidation in the linoleic acid model system (Vijaykrishnaraj et al., 2015, 2016).

**Table 4:** Bioactive compounds contents of the fortified biscuits prepared from wheat–purple rice flour blends mixed with mussel powder and mixed spices

Bioactive compounds	% defatted green‐lipped mussel powder				LSD (5%)
	Control	5	10	15	
Total phenolics (mg GAE/100 g DW)	2664.72 ± 8.68^d^	3120.98 ± 0.78^c^	3311.62 ± 13.77^b^	3522.96 ± 10.87^a^	10.88
Anthocyanin (C3G, mg/kg DW)	19.20 ± 0.10^d^	24.38 ± 0.25^c^	25.88 ± 0.10^b^	27.55 ± 0.25^a^	0.31
Flavonoid contents (mg CE/100 g DW)	1.12 ± 0.07^d^	1.52 ± 0.07^c^	1.77 ± 0.10^b^	2.12 ± 0.10^a^	0.01
DPPH (μmol Trolox/g DW)	47.52 ± 0.11^d^	48.06 ± 0.01^c^	48.62 ± 0.06^b^	48.96 ± 0.07^a^	0.12
ABTS (μmol Trolox/g DW)	11.95 ± 0.18^d^	16.39 ± 0.24^c^	30.60 ± 0.19^b^	41.07 ± 0.03^a^	0.29

LSD, least significant difference.

Each row, sample means not having the same letter are significantly different (Duncan's multiple range test, *p *<* *.05). Values represent mean ± SE.

The antioxidant activities of the biscuits were determined by two different methods: 2,2‐diphenyl‐1‐picrylhydrazyl (DPPH) and 3‐ethyl‐benzothiazoline‐6‐sulfonic acid (ABTS) assays, as shown in Table 4. The highest DPPH activity was exhibited by biscuits containing 15% defatted mussel powder. In general, biscuits made from composite flours had decreased the DPPH activity after the baking process (Kaur et al., 2017). Results by Vijaykrishnaraj et al. (2015) showed that the DPPH activity for raw green‐lipped mussel powder was 36%; however, there was no significant difference in total antioxidant activity between fresh and cooked products. When included at a high level in pasta, antioxidants showed a loss activity (Vijaykrishnaraj et al., 2015). Table 4 also shows the antioxidant activities of the biscuits enriched with different levels of defatted mussel powder determined using the ABTS assay. The 15% defatted mussel powder biscuits also exhibited the strongest ABTS capacity followed by the 10% and 5% defatted mussel powder biscuits respectively.

A study by Vijaykrishnaraj et al. (2015) confirmed that mussel powder could be incorporated in gluten‐free pasta to achieve antioxidant efficacy. According to the results of Abdel‐Samie et al. (2010), antioxidant compounds and the antioxidant activity of biscuits enriched with 5% cumin and ginger showed higher antioxidant effects than the control biscuits. From an antioxidant point of view, the present study indicated that biscuits containing 15% mussel powder contained significant amounts of antioxidant compounds and, consequently, antioxidant activities.

### 3.4 Sensory attributes for preference

A 9‐point hedonic scale method is widely used to evaluate consumers' acceptance of food products by food scientists and technologists. It is effective, used less time than other methods, and can be carried out by either trained or untrained panelists (Sharma, Saxena, & Riar, 2016). The sensory evaluation of biscuits prepared from wheat–purple rice flour that included different levels of defatted mussel powder in the biscuit mix are presented in Table 5. The sensory panelists rated the control biscuits with the highest score (over 7) for oiliness, crunchiness, flavor, and overall acceptability. These attributes were significantly decreased in the biscuits which incorporated different levels of defatted mussel powder. The enrichment with defatted mussel powder up to 15% resulted in a significant increase in hardness (Table 3) compared to the control biscuits. The texture of gluten‐free bread and the acceptance scores improved following the addition of green mussel powder into breads (Vijaykrishnaraj et al., 2016). On the other hand, there were no significant differences (*p *>* *.05) in liking scores for color and overall acceptance among the fortified biscuits and the control biscuits. The defatted mussel powder enrichment significantly decreased the mussel flavor scores of the biscuits; however, the biscuits were still liked slightly by the panelists. The low moisture content in fat‐containing food (biscuits contain 23–24% fat) can accelerate lipid oxidation reactions since the substrates and reactants become more concentrated (Chung et al., 2014). The highest overall acceptability score was for biscuits containing 5% defatted mussel powder and, after this level of substitution, a decrease in acceptability scores was observed; however, there were no significant differences (*p *>* *.05) among the fortified biscuits for all attributes. Vijaykrishnaraj et al. (2016) reported that the sensory evaluation scores were acceptable when 5% green‐lipped mussel powder was added to gluten‐free bread. In this study, biscuits enriched with defatted mussel powder of up to 15% were accepted by panelists. The defatted mussel biscuits developed were nutritionally enriched and would be acceptable by consumers suffering from diabetes.

**Table 5:** Mean liking scores of the fortified biscuits prepared from wheat–purple rice flour blends mixed with mussel powder and mixed spices

Attributes	% defatted green‐lipped mussel powder				LSD (5%)
	Control	5	10	15	
Color	6.42 ± 0.26^NS^	6.19 ± 0.20	6.38 ± 0.24	6.27 ± 0.27	1.86
Overall appearance	6.69 ± 0.23^NS^	5.92 ± 0.29	6.42 ± 0.27	6.46 ± 0.30	1.97
Touch/oiliness	7.27 ± 0.22^a^	6.54 ± 0.26^ab^	6.35 ± 0.25^b^	6.38 ± 0.30^b^	0.87
Crunchiness	7.62 ± 0.20^a^	6.58 ± 0.27^b^	6.46 ± 0.27^b^	6.35 ± 0.27^b^	1.02
Flavor	7.04 ± 0.36^a^	5.46 ± 0.45^b^	5.27 ± 0.36^b^	4.62 ± 0.40^b^	1.52
Overall acceptability	7.42 ± 0.25^a^	5.65 ± 0.40^b^	5.38 ± 0.30^b^	5.31 ± 0.18^b^	1.67

LSD, least significant difference; NS, not significant.

Each row, sample means not having the same letter are significantly different (Duncan's multiple range test, *p *<* *.001). Data are expressed as mean ± SE.

## 4 CONCLUSIONS

Developing novel biscuits by supplementing refined wheat flour with purple rice flour enriched with defatted mussel powder has been investigated by Klunklin and Savage (2018). The biscuits hold high promise with regard to antioxidant compounds and antioxidant activities. Moreover, the water content and hardness were important factors when evaluating biscuit quality without causing adverse effects after adding the defatted mussel powder. Mixed spices and galangal were used to enhance the flavor of the biscuits enriched with the defatted mussel powder and this was successful, based on the liking scores of the flavor attributes measured by the panelists. Therefore, defatted green‐lipped mussel powder was a nutritious, bioactive compound that can be incorporated in biscuit mixes up to the 15% level. The sensory evaluation proved that the addition of defatted mussel powder in the biscuits led to acceptable liking scores at the highest incorporation level. Natural protein from defatted green‐lipped mussel powder can be used as an alternative protein to improve the nutritional values (higher protein, fiber and ash content with lower fat content) of food products and achieved acceptable scores by panelists.

## CONFLICT OF INTEREST

None declared.

## ETHICAL STATEMENT

This study was approved by the Lincoln University Human Ethics Committee (2017‐24).
